# Midnight Oil or Midnight Sleep?

**Published:** 1891-09-19

**Authors:** 


### The Hospital

A Weekly Journal of

Science, Medicine, Nursing, and Philanthropy.

*For Hospitals, Asylums, Infirmaries, Dispensaries, Medical Practitioners, Students, Nurses, and the Charitable Public.*

Vol. X.—No. 260.] [Sept. 19, 1891.

### Midnight Oil or Midnight Sleep?

Professors and lecturers are generally very grave men. Most of us are grave in proportion to the weight we have to carry in life. No weight is so burdensome as the sense of one's own dignity and importance. The college professor is, therefore, to be deeply sympathised with when his looks and behaviour betoken a gravity which is quite preternatural. Some of us remember the professor of a quarter of a century ago; his measured walk, his spectacles, his careful pronunciation, his ancient saws and modern instances. He believed in midnight oil; his students, it is to be feared, too often believed in midnight tobacco and imperial pints of Bass's ale.

Nowadays every man is physiological, or he is nothing. For even a soldier or a school inspector to confess that he knows nothing of physiology is to confess that he is like a six days' old kitten, whose eyes are not yet opened to see the true intellectual light and sun of the end of the century. The physiologist, like Mr. J. L. Toole in "Paul Pry," has his eye on the midnight oil—his eye and his spectacles. What has the physiologist to say to the midnight oil? He has so much to say that he puts down his umbrella, adjusts his coat collar and his wristbands, and prepares himself for a serious half-hour of gravely scientific remonstrance and denunciation.

The brain must work, but it must also rest. The brain that rests best works best and longest. The brain that uses the midnight oil cannot rest. It is like the troubled sea in that it tosses too much when it attempts to sleep. It is also like the troubled sea in that it "throws up a great deal of mire and dirt" when literature happens to be its peculiar work. Midnight oil "came in" long before physiology. The records of its fossil period speak of sublapsarianism and sapralapsarianism, of pre-established harmony and prevenient grace. Fine old definitions and formulisations they were, and served the useful purpose of keeping many inconsequent people amused and employed through the centuries known as the dark ages. But the times have changed, and with their change the human spirit has become at least more energetic and curious. It will not have the ancient definitions at any price.

Physiology is a bold questioner. She had need be bold in such a tradition and custom ridden world as this. What astonishing propositions the metaphysicians and the priests of all ages, from that of Artemis downwards, have insisted that sane men shall believe! Physiology soberly asks for reasons, and insists upon experience. What is her challenge to the trimmers of the midnight lamp? "Show me the fruits of your labours," she says, "the fruits as seen in the work you have produced; and as seen also in yourselves, the workers." The most whimsical of all the conclusions of philosophy are those which have been reached by workers who worked when other men were asleep. The sermons which contain the least admixture of sober sense with the largest expansion of fanaticism and intellectual feebleness are those which smell most of the midnight oil. Men who can go back upon a long and varied experience of college life can remember what musty and pompous inanities those professors were who solemnly advised that work should be continued late as well as early, and should seldom be left off, even at midday!

The earnest medical student is the very last man in the world who should burn the midnight oil. His work is work which does not demand memory only, but thought. The only way by which the mere facts of chemistry and anatomy, for example, can be got to remain in the mind, is to first understand them thoroughly. They cannot be learned by rote as the parrot learns his lessons. They must be taken into the consciousness as clearly comprehended statements and things. But if a man begins at seven o'clock and works on until midnight in the way here described, how is his roused and excited brain to cease working when he goes to bed? It does not cease. The cells continue their activity, the coursing blood, rushing to the brain in response to the demand made for it by the energetic molecules, continues to flow onward through the distended cerebral vessels and makes sleep impossible. One o'clock comes, and the brain cells and blood currents are only beginning to sober down. Two strikes, and an uneasy sleep is stealing over the senses that still even in their unconsciousness strive to master and retain the new facts which have been forced upon the cerebral nerves and their centres. Not perhaps until three in the morning is sound and refreshing sleep obtained. At seven the diligent student is again up, and has his head and body submerged in the stimulating bath. His physique is splendid; and for a time the work and want of sufficient rest hardly seem to tell upon him at all. He feels all the exhilaration and defiance of a successful conqueror.

Is it possible to make the student of three and twenty believe in reckoning days? Hardly! But he will know all about them long before he is three-and-forty. Not even the worried business man knows so well as the hard-worked medical scientist of ripe experience, the unalterable logic of spending and ending.

Physiological resources, although they are very elastic within limits, yet have limits which are so sharply defined, that to step beyond them often means absolute bodily and mental ruin. There is no overstepping of the limit which is more dangerous than that of doing work which curtails sleep. Sound and sufficient sleep is the most indispensable of all the conditions of a sound and efficient brain. The miseries alone of the sleepless man are creditors which the most stoical may dread; his incapacities are such that great work and great success are generally as hopeless for him as the possibility of riding through the air without a balloon or wings. Ten years of such sleeplessness as some men have endured would cure the most ardent medical enthusiast in the world of his passion for the midnight oil.

But if men do not work late and early, how are they to gain the honours which are to distinguish them from the crowd, and to form the starting point of the success of their after lives? A most practical and urgent question, but a question which sober experience is much more likely to answer wisely than youthful eagerness and ambition. The greatest and highest success in life is achieved, like the winning of a long race, by him who has the greatest staying power. What is the best of all the possible kinds of brain for a man who has to follow throughout his life an intellectual calling like that of the higher walks of medicine? It is a brain that is at once clear and strong. Undue and prolonged mental exertion in the student period may give great clearness of intellect; possibly even an abnormal clearness; but it can never give strength. Clearness without strength can no more win in the long and arduous race of life than speed without staying power can win in a foot race of ten miles. Unintelligent and impulsive medical professors—and there are many such—may urge men to competition for the highest college honours, even at the risk of a total breakdown in brain and body. Such professors are among the worst enemies young men can have, and they are among the worst enemies the medical school and the medical profession can have. What the medical profession demands is men of clear and strong intellect, full of practical resources, not mere dilettanti speculators in incomprehensible medical hypotheses. Sublapsarianism and sapralapsarinism have their equivalents and their ridiculous professors in the medicine of to-day as they had in the theology of a long past time. What honest and capable man desires to be a medical acrobat, or a tight-rope dancer? He prefers a less "elevated," though much more useful and honourable kind of occupation.

The day is the time for work: the night for sleep: sleep sound, quiet, and peaceful as death. The learned medical professor tells his students all this in his book or his lecture. But he seldom thinks of asking them to apply his lofty and ideal principles to the details of their own lives. The medical student who attends lectures and hospital work for three or four hours daily, and reads steadily and well for four or five hours more does amply sufficient both for honour and profit. Whatever he may do in the class or the professional examinations he will certainly beat the midnight-oil man in the greater and more prolonged "examination of life." The first thing that the world demands of professors and teachers of all kinds is that they shall practice their own principles. A teacher of physiology who encourages brain work at midnight ought to be considered insane.

